# Geometrically Repatterned Immunological Synapses Uncover Formation Mechanisms

**DOI:** 10.1371/journal.pcbi.0020171

**Published:** 2006-11-10

**Authors:** Marc Thilo Figge, Michael Meyer-Hermann

**Affiliations:** 1 Institute for Theoretical Physics, Johann Wolfgang Goethe University, Frankfurt am Main, Germany; 2 Frankfurt Institute for Advanced Studies, Johann Wolfgang Goethe University, Frankfurt am Main, Germany; Eidgenössische Technische Hochschule-Zürich, Switzerland

## Abstract

The interaction of T cells and antigen-presenting cells is central to adaptive immunity and involves the formation of immunological synapses in many cases. The surface molecules of the cells form a characteristic spatial pattern whose formation mechanisms and function are largely unknown. We perform computer simulations of recent experiments on geometrically repatterned immunological synapses and explain the emerging structure as well as the formation dynamics. Only the combination of in vitro experiments and computer simulations has the potential to pinpoint the kind of interactions involved. The presented simulations make clear predictions for the structure of the immunological synapse and elucidate the role of a self-organizing attraction between complexes of T cell receptor and peptide–MHC molecule, versus a centrally directed motion of these complexes.

## Synopsis

Adaptive immunity is a response of the immune system that involves the activation of lymphocytes and that is most effective in defending against virus-infected cells, cancer cells, fungi, and intracellular bacteria. Central to this response is the interaction between a T cell and an antigen-presenting cell, and in particular the communication of information mediated by the T cell receptor and co-receptors. The contact zone between the cells is a highly organized interface, which is termed the immunological synapse, where both the spatial and the temporal organization of the bound receptors contribute to the generated activation signal on antigen recognition. Although a considerable amount of experimental and theoretical studies have dealt with the immunological synapse, the mechanisms that control its formation are still under discussion. In 2005, Mossman et al. conducted ingenious experiments using nanometer-scale structures to geometrically repattern the immunological synapse. These experiments are reproduced by Figge and Meyer-Hermann applying computer simulations, based on an agent-based model approach, to uncover the emerging structures as well as the underlying formation mechanisms. Clear predictions for the structure of proposed geometrically repatterned immunological synapses are obtained that will further elucidate the role of the involved formation mechanisms.

## Introduction

The recognition of pathogens by the T cells of the immune system relies on antigen-presenting cells (APCs) that process pathogen-derived molecules and present them with major histocompatibility complex (MHC) molecules. The surface of APCs is scanned by T cells that bind to peptide–MHC (pMHC) complexes with their specific T cell receptors (TCRs). This interaction can initiate the dynamic formation of an immunological synapse (IS), which is an adhesive junction with a nanometer scale gap between the two cells [1–3]. Depending on the cellular partners, the IS can adopt different topologies. A fixed plan for a stable common structure does not exist but rather a diversity of structures dictated by the diversity of interacting cells [1].

The prototypical IS matures within minutes into a well-organized structure with a characteristic bull's-eye pattern that may remain stable for hours [2,3]. This pattern is composed of an outer ring, which is referred to as peripheral supramolecular activation cluster (p-SMAC), consisting of bound complexes of the T cell's adhesion molecule leukocyte function–associated antigen-1 (LFA-1) and the APC's intercellular adhesion molecule-1 (ICAM-1). The center of the IS, the central supramolecular activation cluster (c-SMAC), consists of bound TCR–pMHC complexes. The hypothesis that this pattern may enhance and sustain TCR signaling and thus the T cell activation has become a matter of controversy during recent years [4–6]. According to these measurements on naive T cells, TCR signaling occurs primarily at the periphery of the synapse and is ceasing before a c-SMAC has formed. Therefore, the bull's-eye pattern might well be the signature of cell–cell interaction rather than a necessary condition for information processing.

Recently, K. D. Mossman et al. [5] performed in vitro experiments in which the IS between a living T cell and a synthetic surface that acts as an artificial APC was geometrically repatterned. The repatterning of the IS is enforced by inhibiting the movement of TCR–pMHC and LFA-1–ICAM-1 receptor–ligand complexes in the bilayer across artificially imposed nanometer-scale chromium barriers within the synthetic surface. A schematic cross-section representation of the T cell–synthetic APC interface is shown in Figure 1, which indicates the impact of barriers on the molecular organization of ISs.

**Figure 1:**
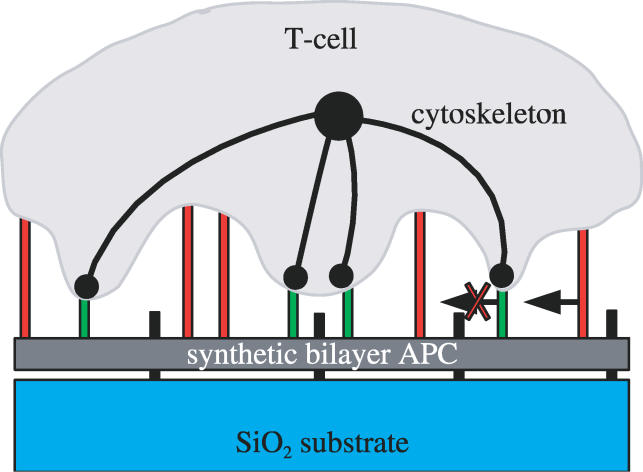
Schematic Cross-Section Representation of the Interface between the T Cell and the Synthetic APC, Based on Figure 1 in Mossman et al. The chromium barriers (black) are implemented in the synthetic bilayer APC and confine the free movement of TCR–pMHC (green) and LFA-1-ICAM-1 (red), as indicated by the crossed arrow. The TCR–pMHC complexes interact with each other via the cytoskeleton of the T cell.

Various aspects of the IS have been successfully analyzed by in silico experiments [7], which allow with relative ease manipulation of each part of a system individually and monitoring of its impact on the system as a whole. Different approaches and theoretical models for the IS are summarized in a recent review [8]. The model for dynamical IS pattern formation by S. Y. Qi et al. [9] is based on a set of partial differential equations and has taken the role of a standard model, which has been frequently used in modified versions as a starting point of later studies [10–13]. Dynamical aspects of the IS pattern formation were recently also studied with an agent-based approach [6,14,15]. In this approach, receptor–ligand complexes are treated as discrete objects (agents) that move and interact with each other on a lattice representing the spatial surrounding. In particular, Weikl et al. [14,15] introduced a model that describes the c-SMAC formation under the assumption of centrally directed TCR–pMHC motion. It is based on a Hamiltonian containing contributions of the elastic energy of the membrane and interaction energies of the receptors, ligands, and glycoproteins. The T cell adhesion dynamics is studied with Monte Carlo simulations, by which thermal shape fluctuations of the membranes are taken into account in a natural way. The model predicts that the final IS pattern with a c-SMAC is only obtained in the presence of active transport processes. These processes are modeled by a constant force acting on TCRs, which is directed towards the center of the contact zone and is attributed to the action of the cytoskeleton.

A different model approach is adopted in the present paper, where we focus on the high potential of geometrical repatterning to uncover the nature of the interaction mechanisms underlying the formation and geometry of the ISs. This is achieved by performing a comparative study of in silico experiments that are based on a generic cellular automaton. In this agent-based approach, receptor–ligand complexes are treated as discrete entities that evolve into a pattern by moving due to thermally induced stochastic motion and according to their mutual interactions.

The experimental basis for these models is given by the observation that, due to large differences in the length of TCR–pMHC complexes (∼15 nm) and LFA-1–ICAM-1 complexes (∼45 nm), elastic membrane forces will drive their segregation [9,11–14]. Since this repulsive interaction acts locally over the distance of the extension of the membrane deformation, this mechanism is not sufficient to explain the fast aggregation and stabilization of TCR–pMHC complexes that form the c-SMAC of the IS. It is well-known that cytoskeletal reorganization of the T cell plays an essential role in this respect [16,17], since TCR activation leads to cytoskeleton activity that feeds back into receptor positioning at the interface of the T cell and the APC. In fact, the formation of the IS is known to depend on an intact cytoskeleton supporting the actively driven process of TCR aggregation [18].

In contrast to previous agent-based models [6,14,15], we do not model the impact of the cytoskeleton by assuming that TCR–pMHC complexes are dragged into a preferred direction. Instead, we propose that the cytoskeleton mediates an isotropic, long-range, attractive interaction between TCR–pMHC complexes that induces the self-organized aggregation of TCR–pMHCs within the cell–cell interface. In addition, the directed motion of TCR–pMHCs is kept as an option, e.g., as the consequence of a specific form of the protein agrin that is expressed in active T cells [19,20]. We implement both mechanisms in the present agent-based model and study their impact on the formation of geometrically repatterned ISs.

It should be noted that in the present model we consider receptor–ligand complexes to move as multimeric units by neglecting the individual unbinding and rebinding of receptors and ligands. As a consequence, we neglect the possibility that receptor–ligand complexes might cross the imposed barriers, which is supported by the experimental observation that stable microclusters are formed and that individual TCRs do not percolate over the barriers [5].

The comparison with existing in vitro experiments on geometrically repatterned ISs reveals that three interaction mechanisms are essential during the synapse formation: (i) adhesion between neighboring TCR–pMHC complexes, (ii) repulsive short-range interactions between TCR–pMHC and LFA-1–ICAM-1 complexes, and (iii) either a centrally directed motion of TCR–pMHC complexes or a long-range attractive interaction between them. To determine the relevant type of TCR–pMHC aggregation mechanism, we propose novel experiments on geometrically repatterned ISs and make quantitative predictions for the occurrence of a pattern transition.

## Results

The in silico experiments are performed for the same geometrically repatterned ISs that were recently studied in in vitro experiments by K. D. Mossman et al. [5]. The T cell lies on the synthetic bilayer APC, and the circular interface has a radius R of approximately 5 μm. As in [9], we use TCR–pMHC and LFA-1–ICAM-1 densities on the order 40 μm^−2^ and 100 μm^−2^, respectively. The receptor–ligand complexes perform random moves within the interface region and interact among each other. The same diffusion constant, D = 0.06 μm^2^/s, is chosen for TCR–pMHC and LFA-1–ICAM-1, which corresponds to a typical value for these membrane-anchored macromolecules [21,22]. We account for the observed TCR–pMHC assembly into microclusters [5] by an adhesive force between direct TCR–pMHC neighbors, and the adhesion strength is characterized by the parameter α. The repulsive, attractive, and directed interactions are characterized by the interaction length L_i_ and the relative interaction strength w_i_ with I = rep, att, and dir, respectively. The details of the cellular automaton are summarized in Materials and Methods.

In Figure 2, the IS pattern formation is presented for the two different types of interactions that both are in persuasive agreement with the experimental findings of [5]. Both simulations account for adhesion between TCR–pMHC complexes and repulsion between neighboring pairs of TCR–pMHC and LFA-1–ICAM-1 due to elastic membrane forces, where the parameter values are the same in both simulations. The only difference between the two simulations lies in the mechanisms for TCR–pMHC aggregation. We consider the cytoskeleton to either mediate a long-range attraction between all TCR–pMHC pairs (see Figure 2A–2H) or model the TCR–pMHC aggregation by an interaction that directs them to the center of the IS (see Figure 2I–2P). The results in Figure 2A–2D and Figure 2I–2L correspond to 30 min of synapse formation, while Figure 2E–2H and Figure 2M–2P show the IS formation at four instants during the first 10 min. From visual judgment we infer that both TCR–pMHC aggregation mechanisms can be reconciled with the experimentally observed geometrically repatterned ISs, since they reveal the same structural correlation in the IS pattern [5]. Furthermore, both simulations also capture the formation of the IS during the first 10 min in agreement with the experiments [5]. During the first few minutes, local microclusters form that may contain roughly 100 TCR–pMHC complexes and that are stabilized by the adhesive force between these complexes (see Figure 2E–2F and Figure 2M–2N). Note that microclusters do not form in the absence of adhesion. In accordance with the experimentally observed time scale, our model describes the migration of microclusters as a whole to the center of the IS, where they coalesce to form a c-SMAC.

**Figure 2:**
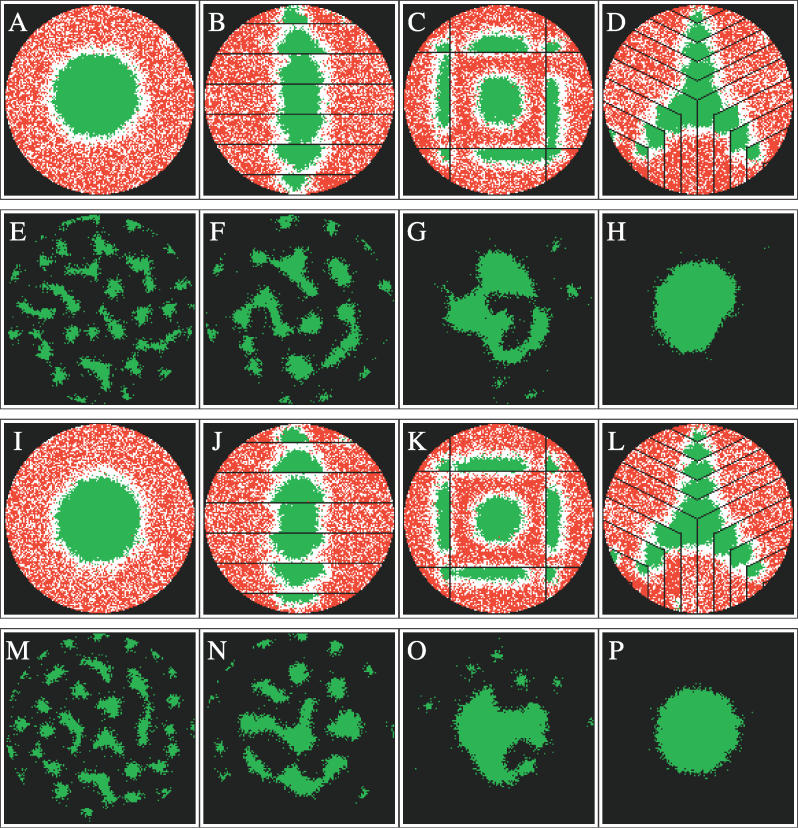
IS Pattern Formation Composed of TCR–pMHC Complexes (Green) and LFA-1–ICAM-1 Complexes (Red) for Two Different Types of Interactions That Both Reproduce the Experimentally Observed ISs (in Mossman et al.) That Were Geometrically Repatterned by Chromium Barriers (Black) Both simulations take adhesion between TCR–pMHC complexes (α = 1), diffusion of TCR–pMHC and LFA-1–ICAM-1 (D = 0.06 μm^2^/s, and short-range repulsion between TCR–pMHC and LFA-1–ICAM-1 (L_rep_ = 0.1R, w_rep_ = −1) into account. (A–H) TCR–pMHC aggregation due to long-range attraction (L_att_ = R, w_att_ = 0.14, w_dir_ = 0). (I–P) TCR–pMHC aggregation due to centrally directed motion (L_dir_ = R, w_dir_ = 3, w_att_ = 0). The IS formation is shown after 30 s in (E) and (M), after 2 min in (F) and (N), after 5 min in (G) and (O), and after 10 min in (H) and (P).

It cannot be excluded that other types of interactions are present, e.g., adhesive forces between LFA-1–ICAM-1 complexes; however, the comparison with the experimentally observed geometrically repatterned ISs indicates that the included mechanisms are sufficient, are all required, and seem to be the most important ones. In addition, depending on the precise interaction parameters, a rich variety of IS patterns is observed. In Figure 3, the results of in silico experiments for the same geometrically repatterned ISs as in Figure 2 are presented with various interaction mechanisms being changed in a stepwise manner. It is confirmed that each of the previously considered ingredients delivers an important contribution to the formation of the geometrically repatterned ISs. In particular, the size of the attraction length L_att_ has a strong impact on the IS pattern. This can be seen in Figure 3A–3D where the simulation results after 30 min of synapse formation are shown for different interaction lengths L_att_, starting from the same simulation parameters as in Figure 2A. It is clearly observed that several TCR–pMHC clusters form in the case of reduced interaction lengths L_att_ < R/2. In the context of immature T cells (thymocytes), multifocal synapse patterns have been attributed to the reduced density of TCRs and thermal fluctuations [10]. However, multifocal synapse patterns are also observed for mature T cells [23]. We find that a reduced interaction length in the attractive long-range interaction between pairs of TCR–pMHC represents a possible explanation for multifocal synapse patterns. Note that the IS patterns in Figure 3B–3D are metastable and may, after an unrealistically long time, still evolve into a bull's-eye pattern by diffusion and coalescence of the clusters.

**Figure 3:**
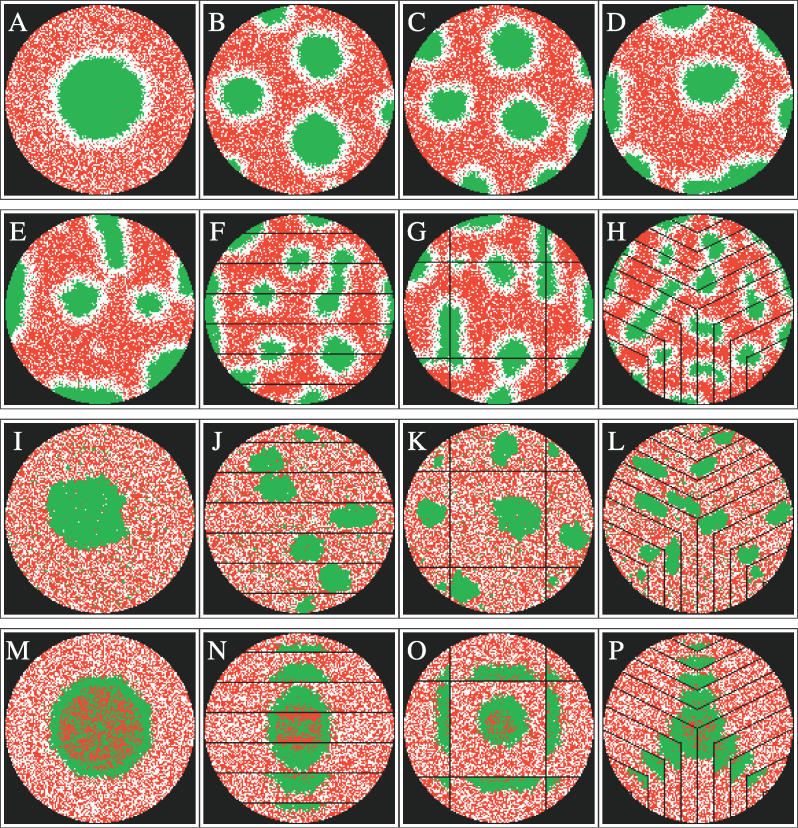
IS Pattern Formation Composed of TCR–pMHC Complexes (Green) and LFA-1–ICAM-1C Complexes (Red) with Various Interaction Mechanisms Being Changed in a Stepwise Manner (A–D) Same parameters as in Figure 2A–2D for varying attraction length: (A) L_att_ = R, (B) L_att_ = 0.43R, (C) L_att_ = 0.29R, and (D) L_att_ = 0.15R. (E–H) Same parameters as in Figure 2A–2D in the absence of long-range attraction (w_att_ = 0) for the geometrically repatterned ISs. (I–L) IS pattern formation in the absence of long-range attraction between TCR–pMHCs (w_att_ = 0) and short-range repulsion between TCR–pMHC and LFA-1–ICAM-1 (w_rep_ = 0), and with strong TCR–pMHC adhesion: α = 5. (M–P) Same parameters as in Figure 2A–2D in the absence of short-range repulsion (w_rep_ = 0).

In Figure 3E–3H, the geometrically repatterned ISs are shown for short-range repulsion between TCR–pMHC and LFA-1–ICAM-1, and for adhesion between direct neighbors of TCR–pMHC complexes, while no long-range attraction as mediated by the cytoskeleton and no directed motion as induced by proteins are taken into account. The simulation time corresponds again to 30 min of synapse formation, and it can be seen that the obtained patterns do not reproduce those observed in the experiment. It is intuitively clear that in the absence of any TCR–pMHC aggregation mechanism a c-SMAC in Figure 3E could only be formed if the TCR–pMHC clusters happen to meet and form larger clusters. Two effects counteract and retard this process: (i) the repulsion between TCR–pMHC and LFA-1–ICAM-1 hinders the coalescence of two clusters, and (ii) the larger the clusters become the slower they move due to the TCR–pMHC adhesion.

An interesting aspect can be observed for the geometrically repatterned ISs in Figure 3F–3H, where TCR–pMHC clusters are found to exist preferentially on opposite sides of barriers. This visualizes the repulsive interaction between TCR–pMHC and LFA-1–ICAM-1 acting across the barrier. Even though a TCR–pMHC cluster that has formed on one side of the barrier cannot cross this barrier, which is implemented in the synthetic APC, it will nevertheless counteract membrane deformations in the T cell and by that favor the accumulation of a TCR–pMHC cluster on the other side of this barrier.

The formation of the bull's-eye pattern is observed if long- and short-range interactions are omitted while adhesion between pairs of TCR–pMHC complexes is increased in strength (see Figure 3I–3L). However, it should be noted that even under these conditions the IS patterns are obtained only after about one day of synapse formation. The unrealistic long simulation time before the bull's-eye pattern emerges in Figure 3I is again related to the fact that large clusters of TCR–pMHC have to be displaced in order to join and form a c-SMAC. In addition, it is found that the bull's-eye pattern only evolves in a narrow region of TCR–pMHC adhesion around α = 5. For α < 5, the emergence of the bull's-eye pattern is prevented by TCR–pMHC diffusion, while for α > 5 its formation time increases by orders of magnitude (unpublished data). It should be noted, however, that even for α = 5 the geometrically repatterned ISs are not in agreement with the experimental observations in [5]. The experimentally observed structural correlations across the barriers in the geometrically repatterned ISs are absent since the various geometric compartments became in fact independent. This stresses once again the requirement of either an attractive long-range interaction between TCR–pMHCs mediated by the cytoskeleton or a centrally directed TCR–pMHC motion induced by proteins to explain the IS formation with its characteristic patterns on a reasonable time scale.

We finally show in Figure 3M–3P the synapse formation after 30 min in the presence of adhesion and long-range attraction (both as in Figure 2A–2D) but in the absence of the short-range repulsive interaction that stems from elastic membrane forces between neighboring TCR–pMHC and LFA-1–ICAM-1 complexes. The in silico experiments reproduce correctly the experimentally observed patterns. However, a complete segregation between receptors of different lengths is not found. Once a swelling outer ring of TCR–pMHC is formed, it becomes increasingly unlikely that it breaks up again to drive more LFA-1–ICAM-1 out of the center of the bull's-eye. In other words, the pattern inversion from an outer TCR–pMHC ring into an outer LFA-1–ICAM-1 ring, which has been observed in the early synapse formation of in vitro experiments [4,18,24], is indicative for the importance of the repulsive interaction between TCR–pMHC and LFA-1–ICAM-1. This statement holds independent of the underlying aggregation mechanism, i.e., long-range attraction between TCR–pMHCs or centrally directed TCR–pMHC motion.

## Discussion

To reproduce the experimentally observed geometrically repatterned ISs by in silico experiments, three relevant interaction mechanisms play an important role: (i) adhesion between neighboring TCR–pMHC complexes, (ii) repulsive short-range interactions between TCR–pMHC and LFA-1–ICAM-1 complexes, and (iii) either a centrally directed motion of TCR–pMHC complexes mediated by aggregation proteins, or a long-range attractive interaction between TCR–pMHC pairs mediated by the cytoskeleton. To answer the question by which aggregation mechanism TCR–pMHCs accumulate at the center of the IS, we propose a conclusive procedure that makes once again use of the high potential of geometrical repatterning experiments. The difference between an attractive long-range interaction and a directed motion of TCR–pMHC can be made visible by realizing that the former interaction depends in a crucial way on the distribution of TCR–pMHC complexes, whereas the latter mechanism is governed by the distribution of proteins. It thus follows that the two mechanisms can be distinguished if the number of TCR–pMHC complexes is geometrically confined in such a way that these mechanisms give rise to clearly distinguishable IS patterns. We propose experiments where the freedom of TCR–pMHC movement is geometrically confined by a barrier that subdivides the IS into an inner and an outer region, respectively, with an inner TCR–pMHC number, N_i_, and an outer TCR–pMHC number, N_o_. Varying the size of the inner compartment is accompanied by a change in the ratio N_o_/N_i_ of the TCR–pMHC numbers in the outer to the inner region. In the presence of directed TCR–pMHC motion, the resulting IS pattern will not change qualitatively as a function of N_o_/N_i_. However, we expect that in the presence of an attractive long-range interaction between TCR–pMHCs, the c-SMAC will only form if N_o_ << N_i_, whereas for N_o_ >> N_i_ the TCR–pMHCs of the inner region will be attracted towards the geometric boundary. To prevent the blurring of the desired effect by the repulsion between TCR–pMHC and LFA-1–ICAM-1 that is acting across the barrier, as has been discussed in connection with Figure 3E–3H, the in silico experiments will be performed for inner regions with linear extensions well above L_rep_.

In Figure 4 the results are presented of two in silico experiments for a circular and a quadratic geometry that subdivide the cell–cell interface into an outer and inner region. These results are obtained for the same parameters that successfully reproduced the experimentally observed ISs in Figure 2, and we checked that all patterns shown after 30 min of synapse formation remain qualitatively unchanged for at least another 30 min (see, e.g., Figure 4C, 4G, 4K, and 4O). In the case of TCR–pMHC aggregation due to the long-range attraction, the IS pattern is seen in Figure 4A–4D to change qualitatively as a function of the radius r of the geometrical barrier. In Figure 4A no c-SMAC is formed after 30 min of synapse formation; instead, the TCR–pMHCs are observed to accumulate at the geometric boundary since they are attracted by the large number of TCR–pMHCs in the outer region. The radius of the circular geometry is r = 0.36R in this case. The pattern is similar for a slightly larger radius r = 0.43R after 30 min, although it seems that a c-SMAC may still develop (see Figure 4B). In Figure 4C, we show for the same radius r = 0.43 R that even after 60 min a clear c-SMAC has not been formed. However, increasing the radius to r = 0.50R, the pattern changes into a c-SMAC, which is clearly visible after 30 min of synapse formation (see Figure 4D). As expected, no pattern transition is observed in Figure 4E–4H for the same parameters in the case of directed TCR–pMHC motion. A similar result is found for the quadratic boundary as a function of the side length s (see Figure 4I–4P).

**Figure 4:**
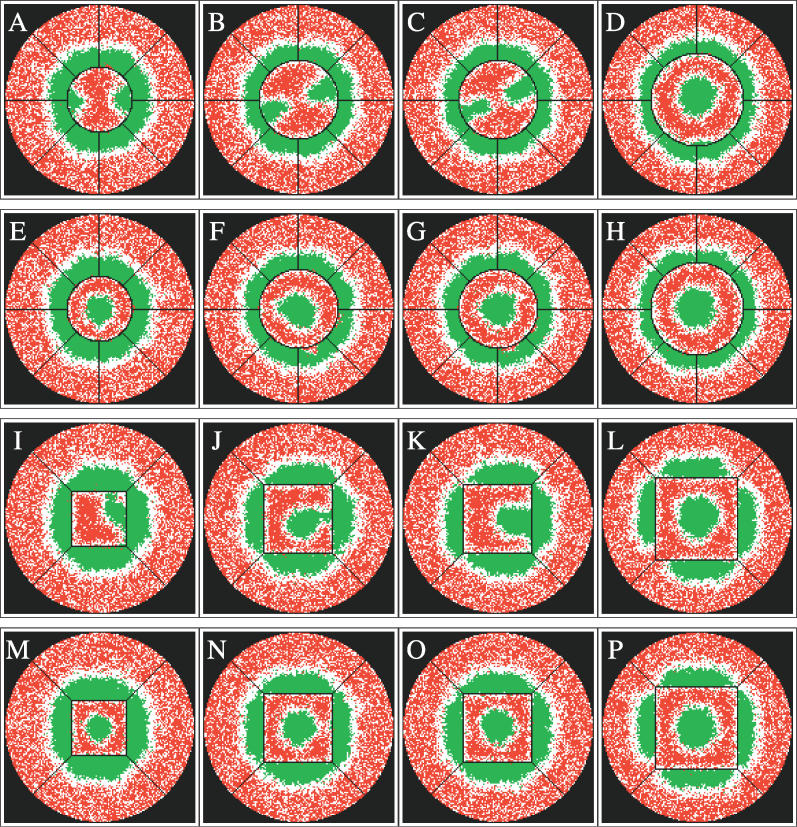
Pattern Transition for Geometrically Repatterned IS: (A–H) with a Circular Geometry and (I–P) with a Quadratic Geometry The parameters are the same as in Figure 2, and TCR–pMHC complexes (green), LFA-1–ICAM-1 complexes (red), and chromium barriers (black) are shown. (A–D) and (I–L) TCR–pMHC aggregation due to long-range attractive interaction (L_att_ = R, w_att_ = 0.14, w_dir_ = 0). (E–H) and (M–P) TCR–pMHC aggregation due to centrally directed motion (L_dir_ = R, w_dir_ = 3, w_att_ = 0). The radius of the circular geometry and the simulation time are (A) and (E) r = 0.36R after 30 min, (B) and (F) r = 0.43R after 30 min, (C) and (G) r = 0.43R after 60 min, (D) and (H) r = 0.50R after 30 min. The side length of the quadratic geometry and the simulation time are (I) and (M) s = 0.57R after 30 min, (J) and (N) s = 0.71R after 30 min, (K) and (O) s = 0.71R after 60 min, (L) and (P) s = 0.85R after 30 min.

A quantitative estimate for the occurrence of the pattern transition is obtained as follows. Assuming the initial random distribution of TCR–pMHCs to be homogeneous, the ratio n_r_ = N_o_/N_i_ is directly related to the areas of the outer and inner regions, respectively, A_o_ and A_i_. The area of the outer region may be expressed in terms of the total interface area A = πR^2^. We then estimate:

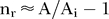
where A_i_ = πr^2^ for the circular geometry and A_i_ = s^2^ for the quadratic geometry. The pattern transition takes place at a critical value of the ratio, n_r_ = n_c_, where n_c_ may depend on geometrical constraints, diffusion, and adhesion, as well as on effects of the repulsive interaction. The pattern transition is found to occur at the critical extensions r_c_ ≈ 0.43R and s_c_ ≈ 0.78R, respectively, for the circular and quadratic geometry. This implies s_c_/r_c_ ≈ π^1/2^ and, thus, that the pattern transition occurs for these geometries at approximately the same critical area for the inner region: A_i_ ≈ 0.2A. Inserting this value for A_i_ into the expression for n_r_ yields a quantitative estimate for the critical ratio:

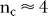
Since this value is roughly the same for both the circular and quadratic geometry, it may be concluded that its deviation from 1 is essentially governed by the residual interactions and not by the constraints of the considered geometries.

It should be noted that, in principle, the IS may be formed by a combination of the long-range attraction and the directed motion of TCR–pMHC. In this case, the transition is expected to be shifted to a larger ratio n_c_ > 4 and thus to a smaller critical value for the area of the inner region, A_i_ < 0.2A. The quantitative estimate for the critical ratio, N_o_/N_i_ ≈ A_o_/A_i_ ≈ 4, may still serve as a guideline for the experimental realization of the pattern transition in the IS formation.

We conclude by once again emphasizing the high potential of geometrical repatterning of ISs with respect to gaining new insight into the underlying mechanisms that govern IS formation. The computer simulations are performed in the classical spirit of an interdisciplinary approach [7], where on the basis of these in silico experiments we propose new in vitro experiments that will advance the understanding of the mechanisms contributing to the IS formation in vivo.

## Materials and Methods

A cellular automaton is used to perform in silico experiments on the formation of geometrically repatterned ISs. To keep the number of involved parameters as small as possible, a minimal phenomenological model is considered where the cell–cell interface is represented by a square lattice of circular geometry with radius R and N sites. To simulate a T cell with a diameter of approximately 10 μm, the lattice constant is set to a = 70 nm and the radius is set to R = 70a, which gives rise to a lattice of circular geometry with roughly N = 15 × 10^3^ sites. Each site has four nearest-neighbors and four (diagonal) next-nearest-neighbors, and can be either empty or occupied by one of the N_TM_ and N_LI_ complexes of TCR–pMHC and LFA-1–ICAM-1 in the system, respectively. The number of receptor–ligand complexes relative to the number of sites that are not excluded by the presence of barriers, are in all simulations fixed around 0.2 and 0.47, respectively, for TCR–pMHC and LFA-1–ICAM-1 [9].

Initially all complexes of TCR–pMHC and of LFA-1–ICAM-1 are distributed randomly on the lattice, i.e., we neglect the recruitment of TCR and LFA-1 from the backside of the T cell since it is known that the cell–cell interface is fully developed during the first 30 s of synapse formation [18]. The time evolution of the system is governed by applying a set of rules at each time step. In practice, we choose Nocc = N_TM_ + N_LI_ sites per time step at random and change the system configuration locally due to the random motion of receptor–ligand complexes and due to their interactions among each other. This procedure is represented by the flowchart in Figure 5 and explained in detail below.

**Figure 5:**
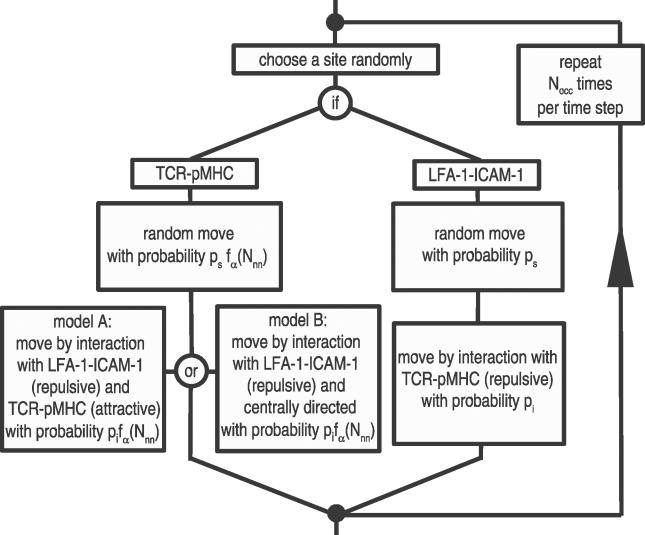
Flowchart of the Cellular Automaton See the text for details.

If a chosen site is occupied, the receptor–ligand complex is allowed to move randomly with a probability p_s_. In the case of LFA-1–ICAM-1, this move is performed if the neighbor site, which is randomly chosen from the eight nearest-neighbor and next-nearest-neighbor sites, is empty. In this procedure, the probability p_s_ for moving to one of the four next-nearest-neighbors is reduced by a factor 2^−1/2^. In the case of TCR–pMHC, whether or not the move is performed depends in addition on adhesive forces between TCR–pMHC complexes at the four nearest-neighbor sites. Adhesive forces are taken into account by an adhesive factor that reduces the probability p_s_ for the move. In the model, the adhesive factor is given by f_α_(N_nn_) = 1/(1 + N_nn_)^α^, where N_nn_ is the actual number of nearest TCR–pMHC-neighbors (0 ≤ N_nn_ ≤ 4), and the parameter α is a measure for the strength of the adhesive force. In all simulations presented in this paper we have chosen p_s_ = 1, from which we estimate the time step for a freely moving membrane-anchored macromolecule with diffusion constant D = 0.06 μm^2^/s to be τ = a^2^/(4D) = 0.02 s.

Furthermore, a randomly chosen receptor–ligand complex may undergo interactions with other receptor–ligand complexes and move according to these interactions with probability p_i_. In the case of TCR–pMHC, this move is again subjected to adhesive forces due to its nearest-neighbor TCR–pMHC complexes. In all simulations presented in this paper we have chosen p_i_ = 0.3, which implies a general dominance of the number of randomly induced moves over the number of moves that are induced by interactions. In other words, the ratio p_i_/p_s_ is comparable to the ratio of the potential to the kinetic energy, and p_i_/p_s_ < 1 has been chosen in the spirit of a fluidity model for the plasma membrane.

Different types of interactions between receptor–ligand complexes are considered. The first type of interaction is related to elastic membrane forces that arise due to the different lengths of TCR–pMHC and LFA-1–ICAM-1 when they are close together. This repulsive interaction of weight w_rep_ is responsible for the segregation of TCR–pMHC and LFA-1–ICAM-1 driving them away from each other if the distance between them is less than the length L_rep_. The distance is related to the region of membrane distortion and is typically on the order of several lattice sites, L_rep_ = 0.1R << R. The second type of interaction gives rise to the aggregation of TCR–pMHC at the c-SMAC of the IS. Two possibilities for the origin of this interaction are considered, which are referred to as model A and model B in Figure 5: (i) the cytoskeleton represents an active source of the central organization of TCR–pMHC. In the model, this is captured by an attractive force between pairs of TCR–pMHC type, which is considered to be long-range in nature with a characteristic length L_att_ (model A); (ii) a centrally directed motion of TCR–pMHC mediated by aggregation proteins that enhance the TCR–pMHC accumulation at a specific point. The interaction range is defined by L_dir_ (model B). In the simulations presented here we either use the interaction of type (i) or (ii) with, respectively, L_att_ = R or L_dir_ = R, if not stated otherwise.

The precise functional dependence of the involved forces is not known and depends on numerous complicated factors, e.g., the time-dependent changes of the membrane under the formation of the IS that have not been monitored in experiments. Thus, we apply the following intuitive rule: if the randomly chosen lattice site is occupied by a TCR–pMHC complex, we calculate the unit vectors in the direction of all LFA-1–ICAM-1 complexes that are less than the distance L_rep_ apart, sum them up, and give this direction a weight w_rep_ < 0 that is related to the strength of the repulsive force. Next, in the case of interaction type (i), we calculate the unit vectors in the direction of all TCR–pMHC complexes that are less than the distance L_att_ apart, sum them up, and give this direction a weight w_att_ > 0 (model A). In the case of interaction type (ii), we calculate the unit vector in the direction of the center of the lattice and give this direction a weight w_dir_ > 0 (model B). In both cases, the two computed vectors are added and the resulting vector is normalized. The latter vector points in the direction of one of its eight neighboring lattice sites, in which the TCR–pMHC complex moves with a probability subjected to the adhesive factor f_α_(N_nn_).

We proceed in a corresponding manner if the randomly chosen lattice site is occupied by an LFA-1–ICAM-1 complex; however, in this case we only have to account for the repulsive force due to membrane distortions by surrounding TCR–pMHC complexes.

In the present in silico experiments, strict barriers are imposed, i.e., receptor–ligand complexes are not allowed to cross the barriers. Related to this issue, at this stage we do not account for the unbinding and rebinding of receptor and ligand, which might induce a small portion of barrier crossing. Furthermore, the recruitment of TCR and LFA-1 from the backside of the T cell during the first few seconds of the IS formation could be taken into account. This would give rise to a time-dependent increase of the TCR–pMHC and LFA-1–ICAM-1 densities at the cell–cell interface that is accompanied by a pattern inversion from an outer TCR–pMHC ring into an outer LFA-1–ICAM-1 ring, as is experimentally observed during early IS formation [4,18,24]. However, in the case of geometrically repatterned ISs, it can be argued that the impact of this effect may be negligible, since experimental observations suggest that barrier crossings are rare events [5], which implies that a time-dependent increase of the receptor–ligand densities may be mainly restricted to the outer region of the geometric pattern of barriers. While these effects may be included in a next developmental step, the charm of the present minimal model is to be simple and at the same time fully appropriate in view of describing the experimentally observed geometrically repatterned ISs.

## Abbreviations

APC: antigen-presenting cell
c-SMAC: central supramolecular activation cluster
ICAM-1: intercellular adhesion molecule-1
IS: immunological synapse
LFA-1: leukocyte function–associated antigen-1
MHC: major histocompability complex
pMHC: peptide–major histocompability complex
p-SMAC: peripheral supramolecular activation cluster
TCR: T cell receptor
